# Factors associated with the persistence of oral 5-aminosalicylic acid monotherapy in ulcerative colitis: a nationwide Norwegian cohort study

**DOI:** 10.1177/17562848211021760

**Published:** 2021-06-28

**Authors:** Reidar Fossmark, Maya Olaisen, Tom Christian Martinsen, Hans Olav Melberg

**Affiliations:** Department of Clinical and Molecular Medicine, Faculty of Medicine and Health Sciences, NTNU - Norwegian University of Science and Technology, Prinsesse Kristinas gate 1, Trondheim, 7491, Norway; Department of Gastroenterology and Hepatology, St. Olav’s Hospital, Trondheim University Hospital, Norway; Department of Clinical and Molecular Medicine, Faculty of Medicine and Health Sciences, NTNU - Norwegian University of Science and Technology, Trondheim, Norway; Department of Gastroenterology and Hepatology, St. Olav’s Hospital, Trondheim University Hospital, Trondheim, Norway; Department of Clinical and Molecular Medicine, Faculty of Medicine and Health Sciences, NTNU - Norwegian University of Science and Technology, Trondheim, Norway; Department of Gastroenterology and Hepatology, St. Olav’s Hospital, Trondheim University Hospital, Trondheim, Norway; Institute of Health and Society, University of Oslo, Oslo, Norway

**Keywords:** 5-aminosalicylic acid, drug persistence, ulcerative colitis

## Abstract

**Background:**

Oral 5-aminosalicylic acid (5-ASA) is the mainstay treatment of ulcerative colitis (UC) and therapy with oral 5-ASA is associated with beneficial outcomes. We have examined factors associated with the persistence of oral 5-ASA treatment in a national cohort of UC patients.

**Methods:**

Patients with newly diagnosed UC from 2010 to 2014 using oral 5-ASA monotherapy were identified by combining data from the Norwegian Patient Registry and the Norwegian Prescription Database. The median follow-up time was 1029 days. Drug persistence was defined as duration of oral 5-ASA preparation as monotherapy. Non-persistence of a oral 5-ASA preparation as monotherapy was defined as stopping oral 5-ASA, initiation of any further anti-inflammatory treatment including a course of glucocorticoids and a change to another oral 5-ASA preparation. Drug persistence was analyzed using the Kaplan–Meier method and influence of covariates on drug persistence was analyzed with the Cox proportional hazard model.

**Results:**

A total of 3421 patients were identified. The overall median 5-ASA drug persistence was 179 days. In univariate analyses, persistence was associated with preparation type and high-dose treatment, while oral glucocorticoid use or hospitalization around the start of oral 5-ASA were associated with shorter 5-ASA persistence. In multivariate analyses, oral glucocorticoids [HR 1.67 (1.54–1.80), *p* < 0.005] and hospitalization around start of 5-ASA [HR 1.23 (1.14–1.34), *p* < 0.005] were associated with non-persistence, whereas high dose (⩾3 g/day) 5-ASA was associated with longer persistence [HR 0.68 (0.65–0.71), *p* < 0.005].

**Conclusion:**

High-dose treatment with oral 5-ASA was associated with longer persistence of oral 5-ASA monotherapy, whereas the presence of factors indicating more severe disease around initiation of 5-ASA monotherapy was associated with a shorter persistence.

## Introduction

5-Aminosalicylic acid (5-ASA) is the first-line therapy for patients with ulcerative colitis (UC) and is effective both for inducing and maintaining remission.^1,2^ 5-ASA is proposed to act through numerous mechanisms, including inhibition of pro-inflammatory mediators and peroxisome proliferator-activated receptor gamma (PPAR-γ) receptor agonism,^3^ as well as altering the intestinal microbiota.^4^

Drug persistence has emerged as a relevant parameter reflecting the long-term therapeutic performance of anti-inflammatory drugs in a real-life setting. Several factors may influence drug persistence, including preparation type, disease severity, prescribed dose and patient compliance. Previous studies focusing on adherence to oral 5-ASA preparations have found young age,^5,6^ single status,^7^ and taking multiple medications as risk factors for non-adherence,^8^ whereas the role of gender seemed equivocal.^7,9^ Possible markers of disease severity such as current glucocorticoid use and pancolitis were associated with higher adherence.^7,9^

5-ASA acts locally in the intestinal mucosa and mucosal 5-ASA concentration has been found to be inversely correlated with disease activity.^10–14^ To avoid absorption and inactivation of unbound 5-ASA in the small intestine,^3,15^ several pharmaceutical delivery systems have been developed to transport oral 5-ASA to the colon. Asacol™, Mezavant™, and Salofalk™ differ to some extent, but all utilize a coating that dissolves at pH > 7, normally occurring in the terminal ileum,^3^ and are often called pH-dependent preparations. Pentasa™ is, on the other hand, a so-called time-dependent preparation, where 5-ASA is coated with a semi-permeable membrane of ethyl cellulose, providing release of 5-ASA from the stomach throughout the gastrointestinal tract.^16,17^ Asacol yields higher mucosal 5-ASA concentrations in the ileum and colon than Pentasa,^10,18^ and Mezavant provides higher mucosal 5-ASA concentrations in the left hemicolon and rectum than Pentasa.^4^ However, a therapeutic mucosal concentration range has yet to be identified^19^; randomized clinical trials (RCTs) aiming to compare oral 5-ASA preparations are scarce and the different 5-ASA formulations are considered clinically equally efficient.^1^ The current study was designed to enable assessment of possible differences in persistence between various oral 5-ASA preparations when used in monotherapy.

The optimal dose of oral 5-ASA treatment has been debated for many years. Meta-analyses of RCTs suggest a threshold for treatment effect at a minimum dose of 2.0 g/day for induction of remission or response,^2^ and patients with moderately active UC may benefit from 4.8 g/day rather than 2.4 g/day.^1,20–22^ Dosing of oral 5-ASA once or twice daily does not seem to affect clinical efficacy.^1^ However, non-adherence to oral 5-ASA, defined as intake of <80% of the prescribed amount, is common in patients with UC in remission outside clinical trials.^7^ Patients who are non-adherent to 5-ASA maintenance therapy are far more likely to relapse than treatment-compliant patients,^8,23^ and it is of clinical interest to identify factors associated with intake of a low dose of 5-ASA.

Oral 5-ASA is the mainstay treatment of UC and population-based studies are useful to identify factors associated with persistence of oral 5-ASA preparations in monotherapy. In the current study, we have examined the persistence of monotherapy with oral 5-ASA preparations in a national cohort of UC patients using a strict definition of non-persistence that included any change in anti-inflammatory treatment.

## Materials and methods

### Data sources

All inpatient and outpatient hospital contacts in Norway are registered in the Norwegian Patient Registry (NPR) and it is mandatory to report diagnoses and clinical procedures. In addition, all prescription drugs sold in Norway are registered by their Anatomical Therapeutic Chemical (ATC) codes as well as preparation name in either NPR and/or for dispensed drugs the *Norwegian* Prescription Database (NorPD). Data from these two registries were combined. The NPR uses unique personal identification numbers from 2008 onwards, which makes it possible to follow individual patients over time.

### Defining UC patients using oral 5-ASA monotherapy

The dataset included every inpatient and outpatient hospital event (at public and private institutions) for all patients who received their first UC diagnosis (ICD-10 code K51) between 1 January 2010 and 31 December 2014, and who were also prescribed an oral 5-ASA preparation after the UC diagnosis. The observation period for UC diagnoses in NPR extended back to 2008 and, for prescription drugs, back to 2004. The time from the first UC diagnosis to initiation of oral 5-ASA could therefore be up to 8 years. Patients who were dispensed oral 5-ASA as the only anti-inflammatory drug registered in NorPD from 3 months after starting oral 5-ASA were identified. Only patients using the four most commonly prescribed oral 5-ASA preparations (Mezavant, Asacol, Pentasa, Salofalk) were analyzed further. Exclusion criteria were the use of an immunomodulator, anti-tumor necrosis factor alpha (TNFα), rectal 5-ASA, or colon surgery (codes as listed in Supplemental Table S1) prior to start of the first oral 5-ASA preparation. The patients were followed retrospectively until 31 December 2017.

### Drug persistence and factors associated with persistence

Oral 5-ASA persistence was defined as the duration of their first oral 5-ASA preparation as a monotherapy. Non-persistence of oral 5-ASA monotherapy was therefore defined as the initiation of rectal 5-ASA or glucocorticoid preparations, systemic glucocorticoids, immunomodulators, anti-TNFα or anti-integrin, change to another oral 5-ASA preparation, or colectomy (Table 1). No dispensal of oral 5-ASA for 1 year was defined as non-persistence, the date of non-persistence was calculated based on the amount that were last dispensed and further consumption of 1.5 g/day, which is one defined daily dose (DDD) as defined by the World Health Organization.^24^ According to the definition of the study population, use of glucocorticoids or rectal 5-ASA during the first 90 days after starting oral 5-ASA, were not recorded as non-persistence, as many patients use several anti-inflammatory medications during a short period of time immediately after the UC diagnosis is established.

**Table 1.** Characteristics of the study cohort of patients with UC starting oral 5-ASA monotherapy.

Preparation	Asacol	Mezavant	Pentasa	Salofalk	Overall
Number of patients [*n* (%)]	1164 (34.0)	986 (28.8)	1106 (32.3)	165 (4.8)	3421 (100.0)
Age at start [years (IQR)]	38 (26–57)	39 (27–57)	38 (26–57)	46 (28–59)	39 (27–57)
Male gender (%)	56.3	53.2	54.3	57.0	54.8
g/d first year (IQR)	2.04 (0.92–3.03)	2.63 (1.38–3.56)	2.13 (0.99–3.18)	2.10 (1.23–3.12)	2.18 (1.05–3.29)
Oral glucocorticoids^a^ (%)	60.1	52.4	60.8	43.0	57.2
Hospitalization^b^ (%)	37.2	32.7	33.4	23.0	34.0
Region in Norway [*n* (%)]					
Central	145 (25.4)	249 (43.7)	146 (25.6)	30 (5.3)	570 (100.0)
North	132 (22.6)	245 (42.0)	191 (32.7)	16 (2.7)	584 (100.0)
South-East	619 (37.7)	369 (22.5)	551 (33.6)	101 (6.2)	1640 (100.0)
West	249 (43.2)	106 (18.4)	205 (35.6)	16 (2.8)	576 (100.0)
Other	19 (37.3)	17 (33.3)	13 (25.5)	2 (3.9)	51 (100.0)

a Oral glucocorticoids 90 days before or after 5-ASA initiation.

b Hospitalization 30 days prior to and 90 days after 5-ASA initiation.

5-ASA, 5-aminosalicylic acid; IQR, interquartile range; UC, ulcerative colitis.

It is known that anti-TNFα use in UC patients differs between the four health regions in Norway.^25^ The hypothetical association between the physicians’ preference for a particular 5-ASA preparation and the clinical threshold for starting additional anti-inflammatory medication was adjusted for at a regional level by analyzing the persistence of oral 5-ASA preparations within each health region.

Disease severity at the time of diagnosis was estimated by two variables that were considered separately as proxies for more severe disease: (1) dispensal of oral glucocorticoids within ±90 days of starting oral 5-ASA. (2) Hospitalization between 30 days prior to, and 90 days after, starting oral 5-ASA.

Oral 5-ASA use/intake during the first year was estimated based on the amount dispensed from pharmacies. High dose 5-ASA was defined as dispensal of ⩾3 g/day during the first year after starting oral 5-ASA.^26^

### Statistics

Variables are presented as mean ± standard deviation (SD) or median [interquartile range (IQR)] depending on distribution. Kaplan–Meier time-to-event analyses were performed to estimate time to treatment discontinuation and comparisons between groups were done using the log-rank test. Univariate and multivariate analyses were carried out using the Cox proportional hazards model to identify factors independently associated with oral 5-ASA persistence; *p* values < 0.05 were considered statistically significant.

### Ethical considerations

The study was approved by the NPR, the Norwegian Data Protection Authority and the Regional Committees for Medical and Health Research Ethics of South-East Norway (2016/113). The dataset does not contain data that can identify individual patients and consent from the patient population was therefore not needed.

## Results

### Patient characteristics

A total of 3421 UC patients satisfying inclusion and exclusion criteria were identified. Patient characteristics are presented in Table 2. Median age at treatment start was 39 (27–57) years and 54.8% of patients were male. Median follow-up time after starting oral 5-ASA was 1029 days.

**Table 2.** Cause of non-persistence of oral 5-ASA monotherapy.

Reason for non-persistence *n* (%)	Overall (*n* = 3421)	Oral glucocorticoid around 5-ASA initiation	
		No (*n* = 1462)	Yes (*n* = 1969)
Glucocorticoid	1063 (31.1)	306 (20.9)	757 (38.6)
Stopped oral 5-ASA	568 (16.6)	331 (22.7)	237 (12.1)
Rectal 5-ASA	437 (12.8)	213 (24.6)	224 (11.4)
Change of oral 5-ASA	409 (12.0)	163 (11.1)	246 (12.6)
Pause >1 year	354 (10.3)	190 (13.0)	164 (8.4)
Immunomodulator	210 (6.1)	43 (2.9)	167 (8.6)
Colon surgery	42 (1.2)	9 (0.6)	33 (1.7)
Anti-TNF or anti-integrin	38 (1.1)	8 (0.5)	30 (1.5)
Persistent at end of observation (no fail)	300 (8.8)	199 (13.6)	101 (5.2)

5-ASA, 5-aminosalicylic acid; TNF, tumor necrosis factor.

### Drug persistence and factors associated with 5-ASA persistence

**Figure 1.**
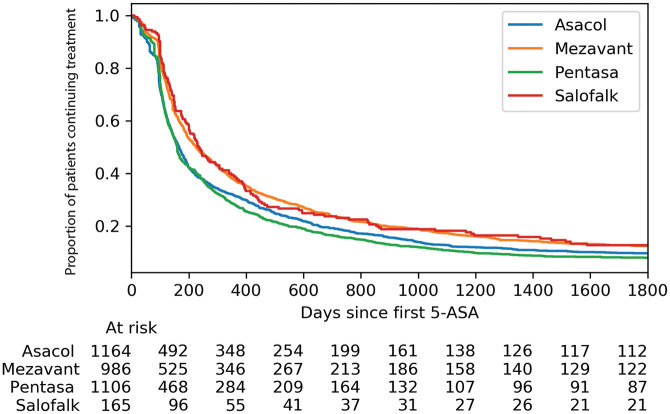
Proportion of patients with ulcerative colitis continuing oral 5-ASA monotherapy stratified by 5-ASA preparation. 5-ASA, 5-aminosalicylic acid.

The median persistence of oral 5-ASA monotherapy was 179 days. The median persistence differed between preparations (*p* < 0.001 for overall difference) in the following order: Salofalk 235 days, Mezavant 226 days, Asacol 166 days, and Pentasa 160 days (Figure 1).

**Figure 2.**
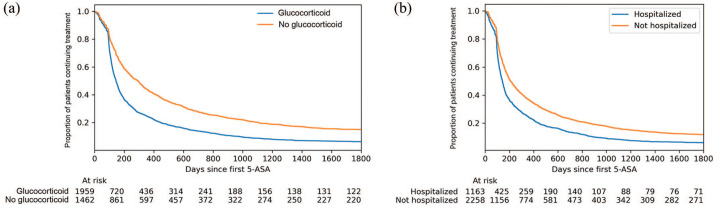
Proportion of patients with ulcerative colitis continuing oral 5-ASA monotherapy stratified by (a) use of oral glucocorticoids ±90 days from starting treatment with oral 5-ASA and (b) hospitalization between 30 days prior to and 90 days after starting oral 5-ASA. 5-ASA, 5-aminosalicylic acid.

Oral glucocorticoids around initiation of oral 5-ASA were prescribed to 57.2% of patients overall. Median 5-ASA persistence in patients using oral glucocorticoids around initiation of oral 5-ASA was shorter than in patients not using oral glucocorticoids (145 days *versus* 293 days, *p* < 0.005) (Figure 2). Oral glucocorticoids were prescribed to a similar proportion of patients using Asacol (60.1%) or Pentasa (60.8%), but to a lower proportion of patients using Mezavant (52.4%) or Salofalk (43.0%). Hospitalization 1 month prior to, or 3 months after, initiation of oral 5-ASA was recorded in 34.0% of the patients overall. Median 5-ASA persistence was shorter in patients hospitalized around treatment start compared with those who were not hospitalized (143 days *versus* 211 days, *p* < 0.005). The proportions of patients with hospitalization around initiation of oral 5-ASA were for Asacol (37.2%), Pentasa (33.4%), and Mezavant (32.7%), and was lower for Salofalk (23.0%).

The median persistence of oral 5-ASA in patients using high dose (⩾3 g/day) during the first year after treatment initiation was longer than in those who did not (193 days *versus* 172 days, *p* < 0.005). The dispensed g/day the first year of 5-ASA treatment was higher in Mezavant users than in patients taking other preparations (Mezavant 2.63 g/day, Asacol 2.04 g/day, Pentasa 2.13 g/day, Salofalk 2.10 g/day).

**Figure 3.**
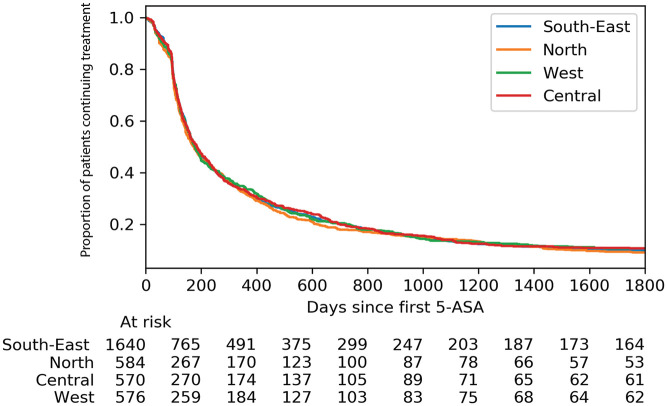
Proportion of patients with ulcerative colitis continuing oral 5-ASA monotherapy stratified by region within Norway. 5-ASA, 5-aminosalicylic acid.

The median 5-ASA persistence differed between the four national regions (*p* < 0.005 for overall comparison), but with only minor numerical differences: Central 184 days, South-East 182 days, West 177 days, North 173 days (Figure 3).

### The cause of non-persistence of oral 5-ASA monotherapy

The major causes of non-persistence were dispensal of further anti-inflammatory treatment as an indication of disease exacerbation, including oral glucocorticoids (31.1%), start of rectal 5-ASA (12.8%), immunomodulator (6.1%), and anti-TNF or anti-integrin (1.1%) (Table 2). A proportion of patients were non-persistent because they stopped (16.6) or paused (10.3%) oral 5-ASA. Stratification for use of oral glucocorticoid around initiation of oral 5-ASA (Table 2) showed that a higher proportion of patients having used oral glucocorticoids around initiation of oral 5-ASA *versus* those who had not, had oral glucocorticoids as the cause of non-persistence of oral 5-ASA (38.6% *versus* 20.9%). A minor proportion (12.0%) of patients had change of oral 5-ASA as cause of non-persistence and the influence of including change of oral 5-ASA or not in the definition of non-persistence is illustrated in Supplemental Figure S1. Excluding change of oral 5-ASA in the definition of non-persistence resulted in a median persistence of 203 days, compared with 179 days when a change to another oral 5-ASA was included.

#### Multivariate analysis

The univariate and multivariate Cox regression analyses included age, gender, preparation type, oral glucocorticoid use, and hospitalization around initiation of oral 5-ASA, dose ⩾3 g/day and region within Norway are presented in Table 3. In the multivariate analysis oral glucocorticoids around 5-ASA initiation [HR 1.67 (95% CI 1.54–1.80)], hospitalization around 5-ASA initiation [HR 1.23 (95% CI 1.14–1.34)], age [HR 1.00 (95% CI 0.99–1.00)], and high dose 5-ASA [HR 0.68 (95% CI 0.56–0.71)] were independently associated with persistence.

**Table 3.** Univariate and multivariate analyses by Cox proportional hazards model identifying factors independently associated with non-persistence of oral 5-ASA monotherapy.

Variable	Univariate		Multivariate	
	HR (95% CI)	*p* value	HR (95% CI)	*p* value
Oral glucocorticoid around 5-ASA initiation	1.61 (1.50–1.74)	<0.005	1.67 (1.54–1.80)	<0.005
Hospitalization around 5-ASA initiation	1.40 (1.30–1.51)	<0.005	1.23 (1.14–1.34)	<0.005
5-ASA preparation (Asacol as reference)				
Mezavant	0.82 (0.75–0.90)	<0.005	0.98 (0.89–1.08)	0.66
Pentasa	1.05 (0.97–1.15)	0.24	1.08 (0.99–1.17)	0.09
Salofalk	0.78 (0.66–0.93)	0.01	0.86 (0.71–1.01)	0.10
Region within Norway (Central as reference)				
North	1.05 (0.93–1.19)	0.42	1.02 (0.90–1.16)	0.69
South-East	1.01 (0.91–1.12)	0.85	1.00 (0.91–1.11)	0.96
West	1.00 (0.88–1.13)	0.99	1.01 (0.88–1.13)	0.96
Age	1.00 (0.99–1.00)	<0.005	1.00 (0.99–1.00)	<0.005
Male	0.93 (0.87–1.00)	0.04	0.93 (0.86–1.00)	0.05
High dose 5-ASA	0.72 (0.69–0.76)	<0.005	0.68 (0.65–0.71)	<0.005

5-ASA, 5-aminosalicylic acid; CI, confidence interval; HR, hazard ratio.

## Discussion

Oral 5-ASA is the standard treatment for patients with mild-to-moderate UC. Oral 5-ASA preparations have high clinical efficacy, few serious side effects, relatively low cost, and optimal use is essential in management of UC. It is therefore of interest to identify factors associated with non-persistence of an oral 5-ASA preparation used in monotherapy. In the current study, we identified 5-ASA preparation type, the dispensed dose, and indicators of disease severity to be associated with persistence of oral 5-ASA monotherapy in univariate analyses.

The various oral 5-ASA preparations have different drug release mechanisms, and previous studies have demonstrated significant differences in colonic mucosal 5-ASA concentrations between preparations. 5-ASA concentrations were lower in patients using Pentasa than in patients using Asacol.^10,18,27^ A recent study found that 5-ASA concentrations in the mucosa of the left hemicolon and rectum were higher in users of Mezavant and high 5-ASA concentrations were associated with a beneficial mucosal bacterial composition.^4^ Furthermore, the mucosal 5-ASA concentration is correlated inversely to the degree of inflammation.^12,14^ In a review of six randomized clinical trials comparing the clinical efficacy of oral 5-ASA preparations 1 year after treatment start, there was no evidence of differences between preparations. However, none of the studies included MMX preparations or compared pH-dependent *versus* time-dependent preparation for doses >2.4 g, and the quality of evidence was considered to be poor.^1^ In the current study we found differences in persistence between 5-ASA preparations in univariate analyses, whereas, in a multivariate analysis indicators of disease severity at treatment start and dose of 5-ASA, but not preparation type, was associated with persistence. Patients taking Mezavant used a higher daily dose (⩾0.5 g/day higher than patients using other preparations) and Mezavant and Salofalk users also had indications of milder disease at treatment start, which could explain the observed differences in persistence between preparations.

The clinical efficacy of oral 5-ASA is dose dependent.^28^ In the current study, we found that patients who were dispensed ⩾3 g/day the first year after treatment initiation had longer drug persistence in univariate as well as in multivariate analysis. This is of clinical interest, as dose is a modifiable factor that potentially could influence treatment outcome. Previous studies suggest that the effective dose of oral 5-ASA is ⩾2 g/day,^29,30^ and it has been debated whether a higher dose has any additional effect. There is evidence supporting that a dose of 4.8 g/day may be beneficial, as patients with moderate UC who received induction therapy with Asacol 4.8 g/day had higher rates of clinical response and short-term mucosal healing than patients receiving Asacol 2.4 g/day.^20,21,31^ Subgroup analyses indicated that 4.8 g/day was beneficial compared with 2.4 g/day in patients with left-sided colitis and in patients with prior glucocorticoid therapy.^21,31^ Additionally, 5-ASA MMX (Mezavant) 4.8 g/day was more effective than 2.4 g/day in patients previously exposed to 5-ASA or an incomplete response after 8 weeks of 2.4 g/day.^32^ A dosage of 4.8 g/day is also more effective for maintaining remission in younger UC patients (<40 years) and in patients with extensive disease compared with 2.4 g/day.^33^ In a network meta-analysis, high-dose ⩾3 g/day was superior to 2–3 g/day for inducing remission, but not for maintaining remission.^26^ The 5-ASA dose dispensed to patients reflects not only the prescription from the treating physician, but actual patient compliance, of which the significance is well documented. Adherence to treatment with oral 5-ASA in UC patients outside clinical trials is within the range of 20–50%.^6,7,34^ Non-adherence is correlated with a 5-fold increased risk of disease flares and may cause a significant proportion of UC exacerbations.^8^ In the current study, patients who were dispensed a 5-ASA ⩾ 3.0 g/day had longer persistence with a 5-ASA monotherapy. The high dose (⩾3.0 g/day) reflects both the prescribed dose and, to some extent, patient compliance, and these findings are supported by previous studies suggesting that a high dose is beneficial in a proportion of patients.

Patients who were hospitalized or dispensed oral glucocorticoids around treatment initiation, as indicators of more severe disease, had shorter 5-ASA persistence than those with less severe disease. This association remained significant also in a multivariate analysis and may partially explain why Mezavant and Salofalk users had longer 5-ASA persistence in univariate analyses. Patients with indicators of more severe disease at the time of starting oral 5-ASA also require additional treatment within a shorter period of time after the first 3 months of initiating oral 5-ASA. The main cause of non-persistence in patients having used oral glucocorticoids around the start of oral 5-ASA was a repeated course of oral glucocorticoids. The evidence supporting early aggressive treatment with biologics is weaker in UC than in Crohn’s disease,^35^ but rapid escalation of treatment in patients with UC has been advocated.^36^

The median persistence was fairly short, which may be related to the relatively broad definition of non-persistence. We also included change to another oral 5-ASA in the definition of non-persistence; however, this was the cause of non-persistence in only 12% of patients. Change to another oral 5-ASA seemed to occur mainly during the first 100 days, and the persistence curves did not separate much further during the remaining observation period.

Strengths of this study include the use of a large and complete national cohort of newly diagnosed UC patients starting oral 5-ASA as well as the long-term follow up. Furthermore, we studied the dispensed dose of 5-ASA, which reflects 5-ASA used by patients better than the prescribed 5-ASA dose. The study is limited by weaknesses inherent to all retrospective studies utilizing data registries, in particular lack of randomization for the variables that were associated with 5-ASA persistence. Furthermore, patient compliance after drug dispensal could not be monitored.

In conclusion, patients with indicators of more severe disease around 5-ASA initiation had shorter persistence of oral 5-ASA monotherapy. High-dose treatment was associated with longer persistence of 5-ASA monotherapy, which is of clinical interest as the dose is a modifiable factor that potentially could influence treatment outcome.

## Supplemental Material

sj-eps-1-tag-10.1177_17562848211021760 – Supplemental material for Factors associated with the persistence of oral 5-aminosalicylic acid monotherapy in ulcerative colitis: a nationwide Norwegian cohort study Supplemental material, sj-eps-1-tag-10.1177_17562848211021760 for Factors associated with the persistence of oral 5-aminosalicylic acid monotherapy in ulcerative colitis: a nationwide Norwegian cohort study by Reidar Fossmark, Maya Olaisen, Tom Christian Martinsen and Hans Olav Melberg in Therapeutic Advances in Gastroenterology
